# Supplementary material for: The relationship between appearance anxiety and depression among students in a medical university in China: a serial multiple mediation model
Source: PeerJ. 2024 Mar 29;12:e17090. doi: 10.7717/peerj.17090 (PMC10984188; doi:10.7717/peerj.17090)
Supplement: Supplemental Information 2 [file peerj-12-17090-s002.docx]

**Categorical Data**

**--A code book to convert numbers**

Basic demographic variables

Gender: 0-female; 1-male

Grade: 1-grade 1; 2-grade 2; 3-grade3; 4-grade4; 5-grade5 or above;

Only a child: 1-yes, 0-no

Home address: 0-Township; 1-City

Monthly income: 0-≤5,000; 1->5,000

Ranking of results:0-Post 50%; 1-top 50%

Single: 1-yes; 0-no

Discretionary income: 0-≤1,500; 1->1,500

the Appearance Anxiety Scale-Brief Version (AASBV): 1-14

5-point Likert scale, ranging from1 (never) to 5 (almost always)

Question 3,4,6,11,12,14 is reverse assignment

the Perceived Social Support Scale (PSSS): 1-12

7-point Likert scale, ranging from 1 (strongly disagree) to 7 (strongly agree)

Family support: ques 3,4 8,11

Friend support: ques 6,7,9,12

Other support: ques1,2,5 10

the SCL-90 Interpersonal Sensitivity Subscale and the Patient Health Questionnaire(PHQ-9): 1-8

1. point Likert scale (0=none 4=critical)

the Patient Health Questionnaire (PHQ-9) to measure depression

4-point Likert scale with a score ranging from 0(none) to 3(always)
